# Relationship between sperm NAD + concentration and reproductive aging in normozoospermia men:A Cohort study

**DOI:** 10.1186/s12894-022-01107-3

**Published:** 2022-10-01

**Authors:** Xueyan Bai, Peng Wang

**Affiliations:** Medical Center for Human Reproduction, Beijing Chao-Yang Hospital, Capital Medical University, Beijing, PR China

**Keywords:** Nicotinamide adenine dinucleotide, Sperm, Age, Sperm quality, DNA fragmentation index

## Abstract

**Background:**

The mechanisms of age-dependent reproductive decline in men are largely overlooked. An age-dependent reduction in nicotinamide adenine dinucleotide (NAD+) levels has been reported in multiple somatic and female reproductive tissues, including oocytes and ovarian tissue. However, the relationship between NAD + levels and male reproduction has not yet been studied. In the current study, the association between sperm NAD + level and paternal age was investigated. In addition, we also investigated whether sperm NAD + levels were related to semen quality.

**Methods:**

In this pilot observational cohort study, semen samples from 51 male subjects who visited a university-affiliated reproductive medical center for preconception consultation (< 30 years: n = 13, 30–40 years: n = 19, > 40 years: n = 19) were recruited. Their anthropometric characteristics were recorded, and semen analysis was performed. Their sperm NAD + levels were evaluated spectrophotometrically.

**Results:**

There were significant differences among the three age groups in the major parameters of semen quality. The sperm NAD + level was, however, similar among the three groups (< 30 years: 91.61 ± 15.59 nmol/10^6^ sperm, 30–40 years: 125.60 ± 16.28 nmol/10^6^ sperm, > 40 years: 115.59 ± 16.55 nmol/10^6^ sperm). Additionally, linear regression also revealed no correlation between sperm NAD + concentration and the age of the participants (r^2^ = 0.018, p = 0.35). Noticeably, a negative correlation was found between the sperm NAD + concentrations and the sperm quality parameters, including sperm concentration (r^2^ = 0.78, p < 0.0001), sperm count (r^2^ = 0.47, p < 0.0001), mobile sperm number (r^2^ = 33, p < 0.0001), and DFI (r^2^ = 0.35, p < 0.0001). The semen volume and mobility rate were not related to the sperm NAD + concentration.

**Conclusion:**

Unlike the age-related decrease of NAD + levels in oocytes and ovarian tissue, the sperm NAD + concentration is not age dependent. Sperm NAD + levels are negatively correlated with sperm quality, suggesting a unique role of NAD + in spermatogenesis, which warrants further study and opens opportunities for pharmaceutical interventions for oligozoospermia.

## Background

In recent years, the trend in parenthood at an older age has become more noticeable, especially in modern societies. Couples intentionally delay their marriage and children bearing to obtain additional education or to build a career. For instance, the average age of first-time fathers rose from 27.4 years in 1972 to 30.9 years in 2017 in the U.S., with 9% of fathers over 40 years old. Such sociodemographic changes have had a profound influence on contemporary fertility.

It is well acknowledged that age-dependent ovarian reserve decrease is a significant factor causing infertility in modern society, as female gamete quality declines more quickly. People have only gradually turned their focus toward the contribution of male aging to infertility. In addition to decreased sexual functioning and coital frequency, it is well established that parental age profoundly impacts male fertility [1]. It is consistently reported that advanced paternal age is associated with prolonged time to pregnancy and decreased chance of pregnancy, even after adjusting for the age of the female partner.

A considerable body of evidence has suggested that semen parameters, including motility, morphology, and volume, are negatively influenced by increased parental age [2]. Notably, in couples receiving assisted reproductive treatment (ART), a meta-analysis also suggested a deleterious effect of increasing male age on the outcome of ART, with clinical pregnancy rates and live birth rates found to be significantly decreased when male age was > 40 years [3].

While most scientific endeavors have focused on female reproductive aging, the mechanisms of age-dependent reproductive impairment in men are largely overlooked. Recent studies have revealed a new marker of female reproductive aging, an age-dependent decline in nicotinamide adenine dinucleotide (NAD+) in oocytes and ovarian tissue [4][5]. Importantly, these studies also revealed a pharmaceutical opportunity: the possibility of restoring NAD + levels by supplementing with NAD + precursors to rescue oocyte quality, embryo development, and functional fertility in aged female mice.

NAD+, the precursor of the pyridine nucleotide family, basically has two functions, a critical electron-transfer intermediate that fuels redox reactions and a substrate for various oxidoreductase enzymes [6]. NAD + has been a popular target for aging studies because of its pivotal role in numerous cellular processes, including DNA repair, cellular energy balancing, epigenetic homeostasis, and gene expression [7]. Studies have shown that environmental stressors can impact the reduction‒oxidation status of NAD + and consequently result in pathological changes in neurodegenerative and cardiovascular diseases [8][9][10]. This coenzyme is commonly found in biochemical pathways and all live tissues, including spermatogenesis tissues [11]. However, the role of NAD + in spermatogenesis and age-dependent reproductive impairment has not yet been well established. Considering the importance and potential therapeutic value of NAD + in female reproductive aging, the relationship between the levels of sperm NAD + and male aging were investigated in this pilot study.

### Methods

## Study population and data acquisition

This study was approved by the Ethics Committee of Beijing Chao-Yang Hospital (Ethical Approval No. 2021-ke-458). All of the participants in the current study were enrolled from the reproductive medical center of Beijing Chao-Yang Hospital from July to September 2021. They were husbands of secondary infertile couples who had a previous birth but failed to conceive after one year of unprotected intercourse. Informed consent was obtained from each participant. Their semen samples were collected for semen analysis as a routine fertility examination. The final 61 participants aged 22–60 years old were all in good mental and physical states. Patients with known conditions that may cause dysspermia were excluded, including chronic diseases, urogenital infections, varicocele, oligozoospermia, and azoospermia. Their general information, including body mass index (BMI), education levels, smoking status, and abstinence period, was collected through a questionnaire.

## Semen collection and analysis

The semen specimens were collected by masturbation after 2–7 days of sexual abstinence. Semen samples were masturbated into a sterile plastic container and then transferred to our andrology laboratory. Incubation at 37 °C for 30 min was performed to allow liquefaction. After liquefaction, the semen volume was evaluated by weighing. A computer-aided sperm analysis (CASA) system (CFT-9200, Ruiqi, Jiangsu, China) was used to measure the semen parameters (sperm count and motility) [12]. A macro spermatozoa-counting chamber (Yuancheng, Nanjing, China) with a 10 μm depth was used to evaluate the semen samples. According to WHO guidelines for each measurement, at least 400 spermatozoa were analyzed [13].

Sperm chromatin integrity was assessed by a flow cytometric-based sperm degree of DNA fragmentation chromatin structure assay (SCSA, CellPro Biotech, Ningbo, China). The assays were performed according to the manufacturer’s instructions. Finally, the DNA fragmentation index (DFI) was used to express the sperm chromatin integrity.

## Measurement of intracellular NAD + levels

Prior to measurement of the sperm intracellular NAD + levels, the semen samples were washed to avoid contamination with seminal fluid. Briefly, a 300 µl semen sample was diluted 1:2 with human tubal fluid (HTF) and centrifuged at 500 × g for 10 min. After carefully aspirating the supernatant, the sperm pellet was resuspended in 1 ml HTF and then centrifuged again at 500 × g for 5 min. The supernatant was carefully removed and discarded. The sperm pellet was used for the measurement of sperm intracellular NAD + levels using an NAD/NADH Cell-Based Assay Kit (Cayman Chemical, MI, USA) according to the manufacturer’s instructions. The sperm pellet was resuspended in 120 µl of the assay buffer provided by the kit and then transferred to a 96-well plate. The following steps of the intracellular NAD + concentration assays were performed strictly according to the manufacturer’s protocol. The NAD + levels were measured and calculated using a plate reader (TECAN Spark, Switzerland) at a wavelength of 450 nm.

### Statistical analysis

Categorical parameters are summarized with counts and percentages. Continuous variables are described with means and standard deviations. First, a one-sample Kolmogorov–Smirnov test was used to determine whether the analyzed variables followed a normal distribution. If the parameter was consistent with a skewed distribution (sperm number, DFI, and NAD + concentration), these variables were log-transformed before further statistical analyses. Second, the chi-square test or Fisher’s test was used to analyze the nominal variables, while Student’s t-test was employed to analyze the quantitative variables. Linear regression was used to explore the correlation between NAD + levels and sperm quality parameters. All data analyses were conducted using SPSS 11.0 software (SPSS Inc., Chicago, IL, USA). A P value < 0.05 was considered statistically significant.

## Results

A total of 61 participants were recruited from October 2020 to January 2021. Ten out of the 61 participants were excluded for conditions including azoospermia (n = 1), oligozoospermia (n = 8), and urogenital infections (n = 1). The mean BMI of the group older than 40 years old was slightly lower than that of the other two groups, but the difference did not reach statistical significance. Approximately 72% of participants had received undergraduate or higher education, and the distribution of education status was similar among the three age groups. Regarding smoking status, approximately 35% of the subjects were smokers, and the percentage of smokers was similar among the age categories. The mean abstinence period was not significantly different among the three age categories (Table 1).

**Table 1:** Characteristics of study participants by age category

Characteristics	All participants		Age category	
		< 30 (n = 13)	30–40 (n = 19)	> 40 (n = 19)
**BMI (kg/m** ^**2**^ **)**				
Underweight (< 18.5)	0	0	0	0
Normal (18.5–24.9)	26	6	8	12
Overweight (25-29.9)	20	5	9	6
Obese (≥ 30)	5	2	2	1
**Education**				
High school and lower	14	4	4	6
Undergraduate and higher	37	9	15	13
**Smoking status**				
Non-smoker	33	8	12	13
Smoker	18	5	7	6
**Abstinence period (days)**				
2–3	19	4	8	7
4–5	22	6	8	8
6–7	10	3	3	4

The semen parameters and sperm kinematics among the three age categories are compared in Table 2. The analyses indicated that there were no significant differences in semen parameters and sperm kinematics between the age categories.

**Table 2:** Comparison of semen parameters and sperm kinematics between age categories

	< 30 years (n = 13)	30–40 years (n = 19)	> 40 years (n = 19)
Semen volume (ml)	2.85 ± 0.42	2.93 ± 0.31	2.97 ± 0.33
Sperm concentration (10^6^/ml)	72.50 ± 7.19	62.64 ± 6.49	60.84 ± 6.63
Total sperm number (10^6^)	233.83 ± 44.15	187.34 ± 33.27	182.81 ± 31.40
Motility rate (%)	34.80 ± 4.00	34.95 ± 2.91	27.66 ± 2.62
Mobile sperm number (10^6^)	70.74 ± 15.39	70.29 ± 14.97	47.73 ± 9.40
DFI (%)	18.75 ± 3.30	22.00 ± 2.14	21.97 ± 3.00

**Fig. 1:**
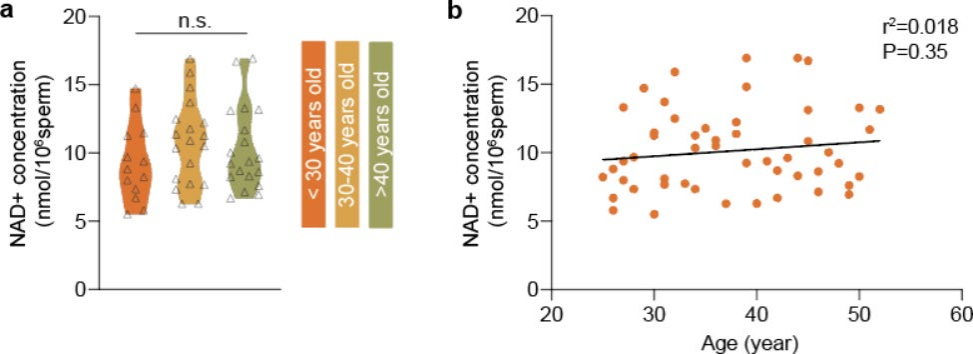
Relationship between intracellular NAD + levels and age (n.s.: not significant)

The relationship between age and NAD + concentration is shown in Fig. 1. The younger group (< 30 years) had the lowest NAD + concentration compared with the two other groups, although the difference did not reach statistical significance. Further linear regression analysis revealed no correlation between the NAD + concentration and the age of the participants.

**Fig. 2:**
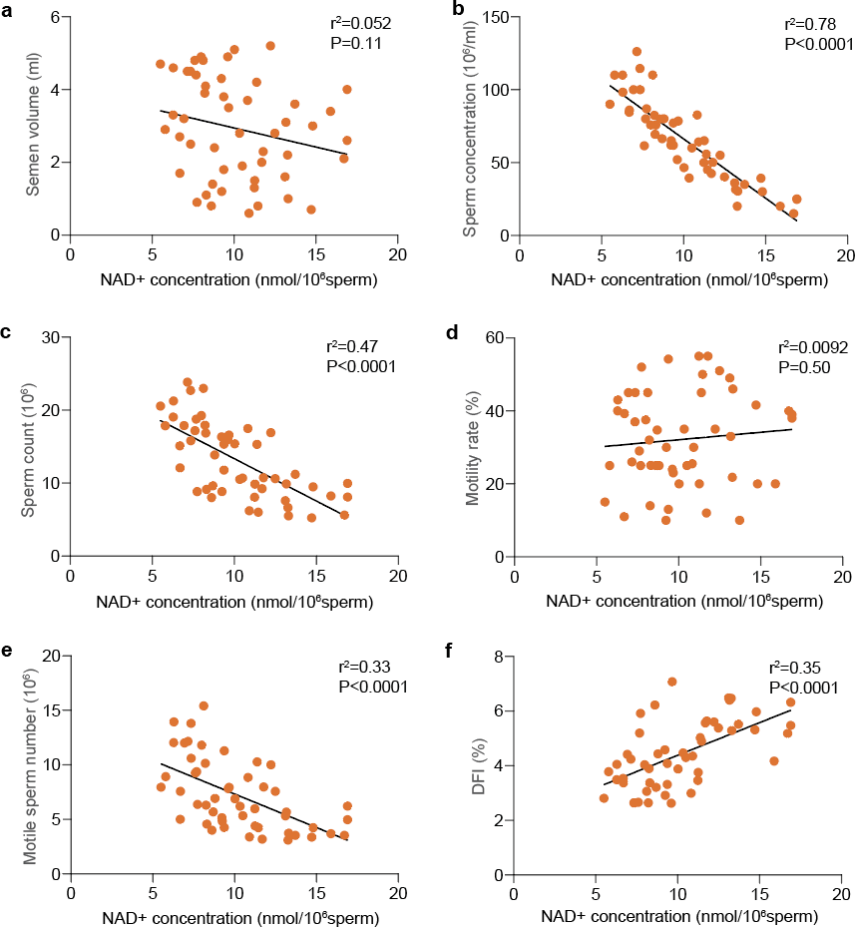
Correlation between intracellular NAD + levels and semen parameters, sperm kinematics, and DFI

Next, linear regression was performed to determine whether the NAD + concentration was correlated with the semen parameters and sperm kinematics. Our results revealed that the intracellular NAD + level was negatively correlated with the sperm quality parameters, as shown in Fig. 2. Additionally, subjects with higher sperm NAD + concentrations also had relatively elevated DFI.

## Discussion

This is the first observational cohort study focusing on the relationship between spermatozoa NAD + levels and reproductive aging in men. Our results revealed no decline in sperm NAD + levels with parental age. Noticeably, the intracellular concentration of NAD + had a significant negative relationship with the sperm quality parameters. In contrast with the well-documented age-dependent decline in NAD + levels during female reproductive aging, our results suggest that NAD + has a different role in spermatogenesis and male reproductive aging.

The age-dependent decline in semen parameters and sperm kinematics has been considered the underlying mechanism of male reproductive aging. Early studies have consistently reported a reduction in sperm motility with advancing age. A 0.17–0.6% decrease in motility per year of age was observed [14][15][16]. For DFI, there has been a good amount of consistent literature showing an increasing rate of fragmentation with increasing age because of increased oxidative stress over time [17][18][19]. Consistent with previous findings, the current study also suggests a tendency toward a decline in sperm quality with age. Our data also support the proposal that advanced age is related to a marginal elevation of DFI.

During the last decade, researchers have revealed the significance of redox-active nucleotides during the process of aging. Evidence has shown that in different somatic tissues, the level of NAD + declines with age. Recent studies clearly indicated that aging is associated with decreased levels of NAD + in rodent oocytes and ovarian tissue, which underlies age-related loss of oocyte function [4]. Importantly, replenishing NAD + by supplementing metabolic precursors reversed ovarian aging by improving mitochondrial functions in aged rodents, which attracted attention as a potential therapeutic target for degenerative diseases [5][20].

To investigate whether the age-dependent NAD + decline also occurs in males, the current study focused on male reproductive aging and measured NAD + levels in human sperm samples. Interestingly, despite our limited sample size, which may increase the chance of a type II error, the results showed no apparent association between age and sperm NAD + levels. This inconsistency in age-related alterations in NAD + levels between oocytes and sperm can be explained by the different self-renewal mechanisms of female and male gametes. According to conventional theory, unlike most somatic tissues, oocytes of mammalian species cannot be regenerated by resident precursor stem cells. The oocyte pool is fixed during *in utero* development, and the oocyte population does not undergo self-renewal, making them highly vulnerable to age-related abnormalities [21]. In stark contrast with oocytes, the male gametes are continuously regenerated throughout adult life in the seminiferous tubules. The entire process of spermatogenesis takes approximately 120 days. Thus, newly generated young germ cells are unlikely to be susceptible to age-related NAD + decline. To clarify the relationship between aging and sperm NAD + levels, large population studies are warranted.

This study reported a negative correlation between intracellular sperm NAD + levels and sperm quality parameters for the first time. Subjects with elevated intracellular sperm NAD + concentrations tend to have poor semen concentration, motility, and DFI. In a recent rodent study, oral supplementation with an NAD + precursor (nicotinamide mononucleotide (NMN)) increased the NAD + concentration in testes and significantly impaired sperm count and motility [22]. This phenomenon can be explained by mitochondrial function. High NAD + levels stimulate mitochondrial biogenesis and function. ROS are a primary byproduct of the NADH-to-NAD + mitochondria conversion through a series of energy metabolism-related processes. As a result, elevated ROS and oxidative stress could result from hyperactivity of the mitochondria. As in sperm, ROS are primarily derived from the mitochondrial NADH-dependent oxidoreductase reaction [23]. The high levels of intracellular NAD + potentially exert deleterious effects on sperm quality and increase DFI by elevating mitochondrial ROS outputs. Additionally, previous findings indicated that supplementation with another NAD + precursor, nicotinamide riboside (NR), downregulates sirtuins, which play a key role in spermatogenesis[24][25][26]. Future clinical trials investigating the effects of oral administration of NAD + supplements on sperm parameters are warranted to answer whether there is a causal relationship between elevated sperm NAD + levels and impaired sperm quality.

## Conclusion

This is the first report focusing on the relationship between the sperm NAD + concentration and reproductive aging in normozoospermic men. The next goal will be to determine whether high levels of sperm NAD + have an adverse influence on the clinical outcomes of ART. The role of NAD + in spermatogenesis also warrants further study, which may lead us to develop novel therapeutic approaches to dysspermia.

## Data Availability

The datasets used and during the current study are available from the corresponding author on reasonable request.

## Abbreviations

ART: assisted reproductive technique.
BMI: body mass index.
CASA: Computer Aided Sperm Analysis.
DFI: DNA fragmentation index.
NAD+: nicotinamide adenine dinucleotide.
NMN: nicotinamide mononucleotide.
NR: nicotinamide riboside.
